# Immune Responses to Circulating and Vaccine Viral Strains in HIV-Infected and Uninfected Children and Youth Who Received the 2013/2014 Quadrivalent Live-Attenuated Influenza Vaccine

**DOI:** 10.3389/fimmu.2016.00142

**Published:** 2016-04-15

**Authors:** Adriana Weinberg, Donna Curtis, Mariangeli Freitas Ning, David Jeremy Claypool, Emilie Jalbert, Julie Patterson, Daniel N. Frank, Diana Ir, Carl Armon

**Affiliations:** ^1^Department of Pediatrics, Division of Infectious Diseases, University of Colorado Denver, Aurora, CO, USA; ^2^Department of Medicine, Division of Infectious Diseases, University of Colorado Denver, Aurora, CO, USA; ^3^Children’s Hospital of Colorado, Aurora, CO, USA

**Keywords:** LAIV, influenza vaccine, HIV infection, children, cell-mediated immunity, ELISPOT, neutralization

## Abstract

The live-attenuated influenza vaccine (LAIV) has generally been more efficacious than the inactivated vaccine in children. However, LAIV is not recommended for HIV-infected children because of insufficient data. We compared cellular, humoral, and mucosal immune responses to the 2013–2014 LAIV quadrivalent (LAIV4) in HIV-infected and uninfected children 2–25 years of age (yoa). We analyzed the responses to the vaccine H1N1 (H1N1-09), to the circulating H1N1 (H1N1-14), which had significant mutations compared to H1N1-09 and to B Yamagata (BY), which had the highest effectiveness in 2013–2014. Forty-six HIV-infected and 56 uninfected participants with prior influenza immunization had blood and nasal swabs collected before and after LAIV4 for IFNγ T and IgG/IgA memory B-cell responses (ELISPOT), plasma antibodies [hemagglutination inhibition (HAI) and microneutralization (MN)], and mucosal IgA (ELISA). The HIV-infected participants had median CD4+ T cells = 645 cells/μL and plasma HIV RNA = 20 copies/mL. Eighty-four percent were on combination anti-retroviral therapy. Regardless of HIV status, significant increases in T-cell responses were observed against BY, but not against H1N1-09. H1N1-09 T-cell immunity was higher than H1N1-14 both before and after vaccination. LAIV4 significantly increased memory IgG B-cell immunity against H1N1-14 and BY in uninfected, but not in HIV-infected participants. Regardless of HIV status, H1N1-09 memory IgG B-cell immunity was higher than H1N1-14 and lower than BY. There were significant HAI titer increases after vaccination in all groups and against all viruses. However, H1N1-14 MN titers were significantly lower than H1N1-09 before and after vaccination overall and in HIV-uninfected vaccinees. Regardless of HIV status, LAIV4 increased nasal IgA concentrations against all viruses. The fold-increase in H1N1-09 IgA was lower than BY. Overall, participants <9 yoa had decreased BY-specific HAI and nasal IgA responses to LAIV4. In conclusion, HIV-infected and uninfected children and youth had comparable responses to LAIV4. H1N1-09 immune responses were lower than BY and higher than H1N1-14, suggesting that both antigenic mismatches between circulating and vaccine H1N1 and lower immunogenicity of the H1N1 vaccine strain may have contributed to the decreased H1N1 effectiveness of 2013–2014 LAIV4.

## Introduction

Influenza causes seasonal disease in temperate climate areas. It is estimated that 5–20% of the population of the US becomes infected with influenza yearly; >200,000 individuals are hospitalized with severe complications; and about 36,000 individuals die annually from influenza-related illness (1). Seasonal influenza vaccines are the mainstay of protection against the influenza and its complications.

The efficacy of the seasonal influenza vaccines depends primarily on the match between the vaccine and the circulating viruses, although heterosubtypic cross-reactivity is well documented (2). In addition, the immune status of the host and the use of live-attenuated or inactivated virus and of adjuvants also contribute to the immunogenicity and efficacy of the influenza vaccines. Influenza vaccines are least immunogenic at the extremes of age and in immune compromised hosts.^1^ The protective effect of inactivated influenza vaccines (IIV) correlates with antibody responses to the vaccines, such that a hemagglutination inhibition (HAI) antibody titer ≥40 is associated with a 50% decrease in the incidence of symptomatic disease in young adults (3). Less is known about other age groups, but HAI titers ≥40 are used as a benchmark for licensure of influenza vaccines.

In children, the live-attenuated influenza vaccine (LAIV) has repeatedly shown increased efficacy compared with IIV against both matched and drifted strains (4–6). Unlike IIV, LAIV does not have a well-established immune correlate of protection, but previous studies showed that cell-mediated immunity (7, 8) and/or mucosal IgA (9, 10) contribute to LAIV-conferred protection. However, these parameters have not been validated and are not currently used to predict the potential effectiveness of LAIV in hosts with different levels of immune competence or in different seasons.

Live-attenuated influenza vaccine has warnings and precautions for HIV-infected and other immune compromised hosts, but its use is not contraindicated in HIV-infected children. There are limited data on the safety, immunogenicity, and efficacy of LAIV in immune compromised hosts and strong concerns that LAIV may cause prolonged viral shedding and serious adverse events in these individuals. We and others showed that HIV-infected and uninfected children have similar amounts and duration of influenza vaccine viral shedding and similar antibody responses to LAIV (11, 12). By contrast, HIV-infected children mount poor antibody responses to IIV (13–16). Efficacy of IIV3 has not been studied in HIV-infected children in the US, but it was poor in HIV-infected children in South Africa (15), making LAIV an attractive candidate for protecting this group from influenza.

We studied the safety and immunogenicity of the quadrivalent LAIV (LAIV4) in HIV-infected children and adolescents compared with uninfected controls vaccinated between August and November of 2013. In the 2013–2014 influenza season, the circulating influenza A H1N1 (H1N1-14) had multiple mutations compared with the influenza A H1N1 in the vaccine (H1N1-09), including a mutation in an HA epitope targeted by human neutralizing antibodies (17–20). In addition, the H1N1 component in LAIV4 was temperature-sensitive so that its immunogenicity was greatly reduced in lots exposed to environmental temperatures ≥80°F.^2^ This translated into lower effectiveness of the 2013–2014 LAIV4 against H1N1 in the US, when most of the vaccine was shipped in late summer and early fall, but not in Canada where the vaccine was shipped later in the season (see text foot note 2). 2013–2014 LAIV4 had excellent efficacy against B Yamagata (BY), the other influenza strain that caused disease in 2013–2014 in the US and elsewhere. The LAIV4 used in our study was shipped directly from MedImmune in August of 2013.

We previously reported that LAIV4 was not associated with any serious adverse events and that viral shedding and antibody responses did not differ between HIV-infected and uninfected participants (21). Here, we report cellular responses to LAIV4, including effector T-cell and memory B-cell responses, in these children and a more detailed analysis of their antibody responses, including differences to the H1N1-09 with H1N1-14 and with BY.

## Participants and Methods

### Study Design

The study planned to enroll 110 subjects who were 2–25 years of age between July and October 2014 with the goal of retaining ≥100 participants for ≥42 days post-immunization. The study was reviewed and approved by the institutional review board. Participants and/or legal guardians provided informed consent and/or assent. Inclusion criteria for HIV-infected participants were CD4 >15% or 200 cells/μL if receiving combination anti-retroviral therapy (cART); or >25% or 500 cells/μL if not on cART. There were no HIV plasma RNA criteria for enrollment.

At entry, participants received a dose of LAIV4 containing live-attenuated influenza A H1N1 California 2009, A H3N2 Victoria 2011, B Massachusetts 2012 Yamagata lineage and B Brisbane 2008 of Victoria lineage. Nasopharyngeal samples for influenza IgA antibodies were collected using flocked swabs (Copan) pre-vaccination and on study days 2–5, 7–10, and 14–21. Blood was obtained pre-vaccination and at 14–21 days.

### Propagation of Influenza Viruses in Embryonated Eggs

A clinical H1N1-14 virus was isolated in Madin-Darby canine kidney (MDCK) tissue culture tubes as previously described (3). The virus stock, consisting of the tissue culture supernatant, was expanded by inoculation into the allantoic cavity of 11-day-old specific pathogen-free (SPF) embryonated hen eggs (Charles River Laboratories, North Franklin, CT, USA) and grown at 37°C for 48 h. The allantoic fluid was harvested after cooling eggs overnight at 4°C, tested for hemagglutinating activity, and stored at −80°C in multiple aliquots. The 50% tissue culture infectious dose (TCID_50_) for each virus was determined by serial dilution of virus in MDCK cells. BY and H1N1-09 viruses were obtained from the influenza reagent repository at ATCC and expanded in embryonated eggs as above.

### HA Gene Sequencing

Viral RNA from H1N1-09 and H1N1-14 was isolated using the QIAamp Viral RNA Mini Kit (Qiagen, Germany). RNA concentration was measured via the Nanodrop ND-1000 Spectrophotometer (Thermoscientific, MA, USA). Subsequently, 1 μg of isolated RNA was used for cDNA synthesis using Superscript II (Invitrogen, Life Technologies, CA, USA). Briefly, RNA samples were incubated at 65°C for 5 min with 1 μL dNTPs (10 mM each) (Invitrogen, Life Technologies, CA, USA) and 1 μL of random hexamers (Invitrogen, Life Technologies, CA, USA). Following that, a mastermix of 4 μL of 5× first strand buffer, 1 μL of DTT (Invitrogen, Life Technologies, CA, USA), and 1 μL of RNAseOUT (Invitrogen, Life Technologies, CA, USA) was added, then incubated at 25°C for 2 min. Then, 1 μL of SuperScript II Reverse Transcriptse enzyme (Invitrogen, Life Technologies, CA, USA) was added, and incubated at 25°C for 10 min, 42°C for 50 min, and finally 70°C for 15 min. In order to analyze mutations located within the HA gene, we designed and employed two internal primers for PCR amplification and sequencing of a 500 base fragment flanking amino acid residue 166: HA280F (5′-TCCTACATTGTGGAAACATCT-3′) and HA780R (5′-TTCGAATGTTATTTTGTCTCC-3′). PCR amplification (ABI 2700, Applied Biosystems, CA, USA) used a protocol of 94°C for 6 min, followed by 40 cycles of: 94°C for 30 s, 53°C for 30 s, 72°C for 1 min 20 s, and a final extension of 72°C for 10 min. PCR products were visualized via electrophoresis in a 2% agarose gel stained with ethidium bromide. PCR products were gel purified and concentrated using the Zymo DNA Clean and Concentrator-5 kit (Zymo, Irvine, CA, USA). Sanger DNA sequencing of PCR amplicons was performed at the DNA Sequencing Service Center at the University of Colorado School of Medicine.

### HAI Titers

Assays were performed as previously described (22) using antigens from the Investigator Reagent Resource, Centers of Disease Control. Blood was collected in heparinized tubes. Plasma was separated by centrifugation and stored in ≥2 aliquots at −20°C. On the day of the assay, plasma was incubated at 1:4 in receptor-destroying enzyme solution (Denka Seiken Co. Ltd.) overnight at 37°C followed by 30 min at 56°C and subsequently with turkey red blood cells (TRBC) at 4°C for 60 min to remove non-specific hemagglutinins. Serial twofold dilution of plasma in PBS starting at 1:10 were mixed with 4 HA units (HAU) of H1N1 CA 2009, H1N1 2014 clinical isolate and B MA 2012 antigens, and TRBC in 96-well V-bottom microtiter plates (Corning) for 30 min at room temperature. The HAI titer was defined as the reciprocal of the last plasma dilution with no HA activity. A titer of 5 was assigned to samples in which the first dilution was negative. Each run included high- and low-titer positive controls. Assays were considered valid if the control HAI titers had less than twofold differences from their previously established mean titers.

### IgA ELISA

Thermo Scientific Immulon II HB microwell strips (Thermo Scientific) were incubated overnight at 4°C on a shaker with each influenza vaccine antigen diluted 1:10 in PBS. Strips were then washed with 0.05% Tween 20 in PBS, blocked with 0.5% fetal calf serum (FCS) in wash solution for 2 h at room temperature, and washed. An aliquot of the M6 transport medium (Remel) in which the nasopharyngeal swabs were collected was diluted at 1:2 and 1:20 (up to 1:300 if needed) in PBS and incubated in duplicate wells/virus for 2 h at room temperature. Strips were washed, incubated with biotinylated goat anti-human IgA (Mabtech 3830-4) 1:1000 in PBS for 1 h at room temperature; washed; and incubated with streptavidin–alkaline phosphatase (Mabtech 3310-8) 1:1000 in PBS for 1 h at room temperature. After washing, bound antibodies were revealed with *p*-nitrophenyl phosphate and read at 490 nm with a Thermo Scientific Multiskan FC ELISA reader (Thermo Scientific). The IgA antibody concentration was calculated by interpolation on the standard curve spanning from 0.08 to 50 μg/mL of the Human-IgA Elisa kit (Mabtech 3830-1AD-6) as per the manufacturer’s instructions. Results were expressed in ELISA units (EU)/mL. Nasal swabs that were below the limit of detection were assigned a value of 0.01 EU/mL. Positive and negative controls were used to validate each run.

### Neutralizing Antibodies

An ELISA-based microneutralization (MN) was performed as previously described (23, 24) on plasma collected at baseline and post-dose 1. Briefly, serial twofold dilutions of plasma starting at 1:10 in phosphate-buffered solution (PBS) were added in duplicate to 100 TCID_50_ of H1N1 CA 2009 or H1N1 2014 clinical isolate and 1.5 × 10^4^ MDCK cells in 96-well microtiter plates. Cell control and virus control wells were included on each plate. After 18–22 h of incubation in a humidified atmosphere at 37°C and 5% CO_2_, the cell monolayer was fixed, and the ELISA was performed using 100 μL anti-influenza A NP mouse antibodies (Millipore, cat. # MAB8257 & MAB8258) followed by goat anti-mouse IgG conjugated to horseradish peroxidase (HRP) secondary antibodies (KPL, cat. # 074-1802). Bound antibodies were revealed with 3,3′,5,5′-tetramethylbenzidine (TMB) substrate. The optical density (OD) was measured with a Multiscan FC ELISA reader (Thermo Fisher) using a 450 nm filter. The neutralizing titer was calculated using the following equation: [median OD of virus control wells + median OD of cell control wells]/2. Samples with discordance larger than 1 dilution between replicates were repeated.

### IFNγ ELISPOT Assay

Peripheral blood mononuclear cells (PBMCs) were cryopreserved as previously described (25) and stored at ≤−150°C. On the day of the assay, cells were thawed slowly as previously described (25). ELISPOT assays were performed using commercial ELISpot^Plus^ kits (MabTech) as per manufacturer’s instructions. Thawed PBMC were allowed to sit overnight and resuspended at 10^6^ PBMC/mL, in RPMI 1640 with glutamine (Gibco), 10% human AB serum (Gibco), 1% penicillin and streptomycin, and 1% HEPES buffer. PBMC preparations with viability ≥70%, as measured by flow cytometry using the Guava easyCyte 8HT instrument (Millipore), were used in these assays. Although viability <70% may decrease the results of functional assays, assay results are stable and unaffected by the viability ≥70 (25–27). Based on our assay optimization results, cells were stimulated in duplicate wells with 4 HAU/well of H1N1 CA 2009, H1N1 2014 clinical isolate, and B MA 2012; 5 μg/mL phytohemagglutinin (PHA; Sigma) or unstimulated control. After a 48-h incubation at 37°C in a humidified 5% CO_2_ atmosphere, plates were washed and stained with anti-IFNγ mAbs as per manufacturer’s instructions. Spot forming cells (SFC) were revealed with colorimetric substrate and counted with an Immunospot S5 UV Analyzer (Cell Technologies Ltd.). Results were expressed in SFC/10^6^ PBMC after subtracting the SFC in unstimulated control wells from those enumerated in antigen- or PHA-stimulated wells.

### IgG/IgA FluoroSpot

Cryopreserved PBMCs were used for the detection of IgG/IgA secreting cells (SC). Cells were thawed, counted, and then stimulated in RPMI 1640 (Gibco) with 10% fetal bovine serum (Gemini Bio-Products), 0.4% penicillin and streptomycin, 1% HEPES buffer (Corning Cellgrow), 1 μg/ml of the toll-like receptor agonist R848, and 10 ng/ml IL-2 for 96 h at 37°C, 5% CO_2_, as per conditions previously optimized in our laboratory. PBMCs with viability ≥55% were used at 250,000 cells/well in duplicate wells of the FluoroSpot IgG/IgA kits (Mabtech Inc.). Assays were performed as per manufacturer’s instructions using H1N1 CA 2009, H1N1 2014 clinical isolate, and B MA 2012 antigens at 4 HAU/well, to coat the plates and an ImmunoSpot II (Cell Technologies Ltd.) instrument to count the IgG and the IgA SC. Results are expressed as SC/10^6^ PBMCs.

### Statistical Analysis

Groups were compared with Yates-corrected chi-square test or Fisher exact test for binary or categorical variables, and the Wilcoxon rank-sum test for continuous variables. Correlation analyses used the Pearson correlation test. Entry nasal IgA concentrations were compared with peak response, which occurred 7–10 days (Visit 3) after immunization for H1N1-09 and H1N1-14 and at 14–21 days (Visit 4) for BY. Analyses were performed using SAS version 9.4 (SAS institute) and Prism version 6 (GraphPad). *P*-values <0.05 were considered significant.

## Results

### Characteristics of the Study Population

The study enrolled 46 HIV-infected and 56 uninfected participants who received LAIV4 (Table 1). HIV-infected and uninfected participants were similar in gender distribution and prior influenza vaccination, but differed in age (medians of 18 and 10 years, respectively) and proportion of blacks (47 and 16%, respectively). The HIV-infected subjects had median CD4 cell numbers of 645 cells/μL and median plasma HIV RNA of 20 copies/mL. Thirty-eight (84.4%) were on cART.

**Table 1:** **Demographics and HIV disease characteristics of the study population**.

Patient Characteristics	HIV-infected children	HIV-uninfected children	*P*-value^a^
	*N* = 45^c^	*N* = 55^c^	
Age in years: median (IQR)	18 (10–23)	10 (7–14)	0.002
Age < 9 years: *n* (%)	10 (22.2)	22 (40.0)	0.11
Median (IQR)	7 (5–8)	6 (4–7)	0.44
Male sex: *n* (%)	28 (62.2)	30 (54.5)	0.50
Black race: *n* (%)	21 (46.7)	9 (16.4)	
White race: *n* (%)	19 (42.2)	45 (81.8)	<0.001
Other/unknown race: *n* (%)	5 (11.1)	2 (3.6)	
Hispanic/latino ethnicity: *n* (%)	7 (15.6)	13 (23.6)	0.48
Received influenza vaccine during 2011–2012 influenza season^b^: *n* (%)	36 (80.0)	43 (78.2)	0.88
Received influenza vaccine prior to 2011–2012 influenza season^b^: *n* (%)	25 (55.6)	27 (49.1)	0.59
CD4+ cell count/mm^3^: median (IQR)	645 (550–995)		
HIV RNA, copies/mL: median (IQR)	20 (1–940)		
Taking cART at study entry: *n* (%)	38 (84.4)		

*^a^Yates-corrected chi-square test or Wilcoxon rank-sum test or Fisher exact test*.

*^b^Number and percent of “Yes” responses, missing entries treated as “No.”*

*^c^Of the 46 HIV-infected participants, one did not receive vaccine and was excluded from the analyses. Of the 56 HIV-uninfected participants, one was lost to follow up after the first visit and was also excluded from the analyses*.

### HA Genotypes of Influenza A H1N1-09 and H1N1-14

The HA amplicon sequences of the H1N1-09 and H1N1-14 strains used in the immunologic assays were 100% identical to the published HA sequences from these two viruses (GenBank accession FJ969540 and KM409098, respectively). The H1N1-14 HA sequence differed from the published H1N1-09 sequence by nine base mismatches over a length of 500 nucleotides. The H1N1-14 HA sequence encoded the K166Q amino acid substitution along with five other non-synonymous mutations (D100N, S188T, S206T, D225G, and R226Q); all other base mutations were silent.

### T-Cell Responses to H1N1-09, H1N1-14, and BY in HIV-Infected and Uninfected Children after LAIV4 2013/2014 Administration

**Figure 1:**
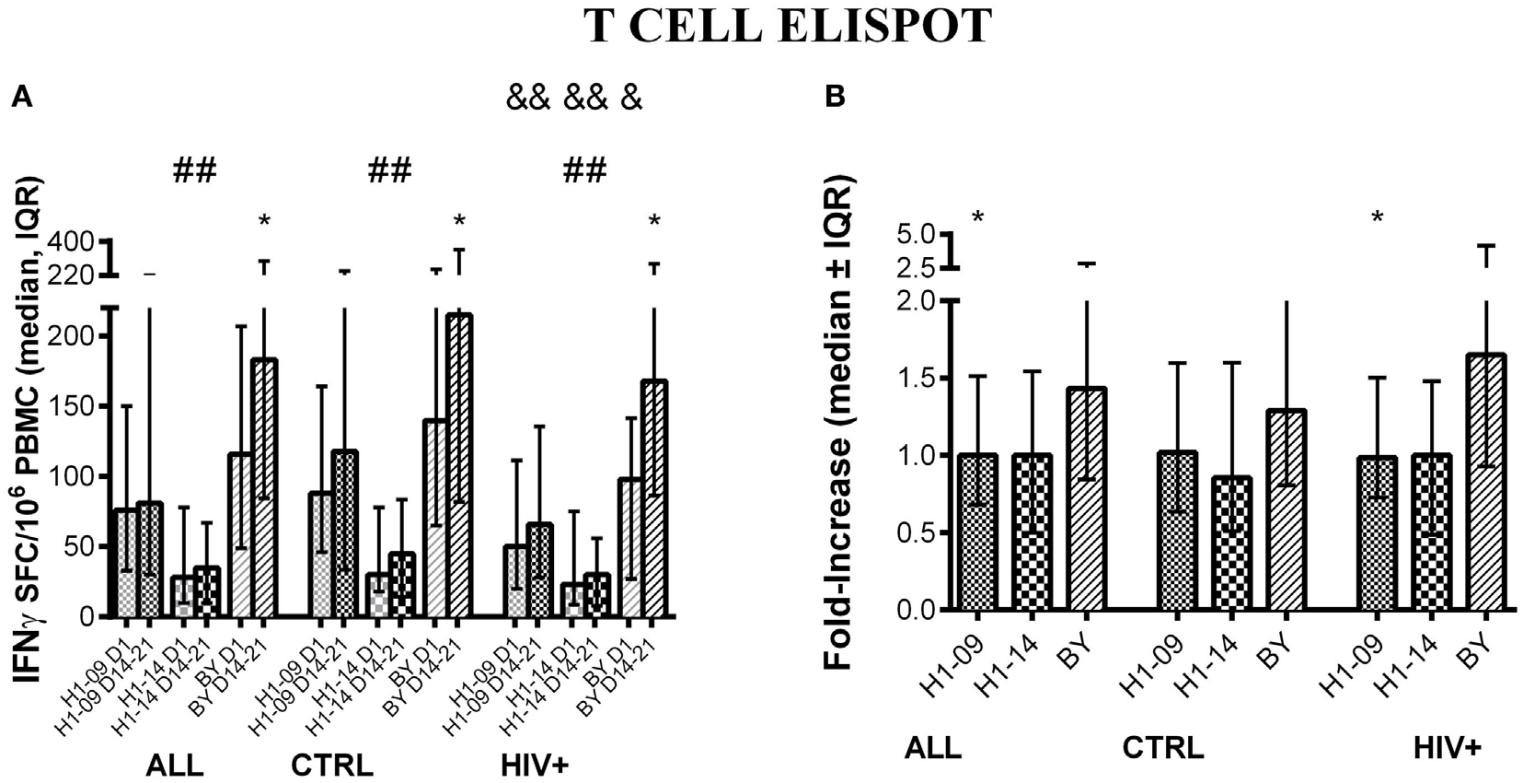
**T-cell responses to LAIV4 measured by ELISPOT**. The data were derived from 45 HIV-infected and 55 uninfected individuals. **(A)** Absolute responses in each group of participants, as indicated below the abscissa, at V1 (before vaccination) and V4 (14–21 days after vaccination) and against the viruses indicated in the labels of the abscissa. Asterisks (*) indicate significant differences between pre- and post-vaccination. Hash tags (#) indicate significant differences between H1N1-09 and H1N1-14. And symbols (&) indicate significant differences between HIV-infected and uninfected participants. **(B)** Fold-increases from pre- to post-vaccination. Asterisks (*) indicated significant differences between H1N1-09 and BY.

Before vaccination (V1), there was a significant difference between H1N1-09 and H1N1-14 effector T-cell responses across all participants [median (inter-quartile range = IQR) of 76 (33; 150) vs. 28 (10; 78) SFC/10^6^ PBMC; *p* < 0.0001; Figure 1A]. The vaccine did not induce a detectable increase at visit 4 (14–21 days) in responses to the H1N1 viral strains and the difference between H1N1-09 and H1N1-14 noted before persisted at visit 4 after vaccination [median (IQR) of 82 (30; 222) vs. 36 (10; 68) SFC/10^6^ PBMC; *p* < 0.0001; Figure 1A]. Vaccination resulted in a significant overall increase of the T-cell responses to BY [median (IQR) of 116 (49; 207) at entry to 184 (86; 296) SFC/10^6^ PBMC 14–21 days after vaccination; *p* < 0.0001; Figure 1A]. The SFC/10^6^ PBMC fold-increase from baseline to 14–21 days post-vaccination was significantly higher for BY compared to H1N1-09 (*p* ≤ 0.002; Figure 1B).

Compared with HIV-uninfected, HIV-infected participants had lower ELISPOT responses to all influenza strains before vaccination (*p* ≤ 0.06; Figure 1A) and lower responses at 14–21 days after vaccination to H1N1-09 and H1N1-14 (*p* ≤ 0.04; Figure 1A), but not to BY (Figure 1A). The fold-increase from pre- to post-vaccination did not differ by HIV status (Figure 1B). Age did not have a significant effect on T-cell responses: HIV-infected participants <9 years of age had 1.1 and 1.3 median fold-increases to H1N1-09 and BY, respectively, while HIV-infected participants ≥9 years of age had 1.0 and 1.7, respectively. Corresponding numbers for HIV-uninfected participants were 1.1 and 1.3 and 1.0 and 1.4.

### B-Cell Responses to H1N1-09, H1N1-14, and BY in HIV-Infected and Uninfected Children

**Figure 2:**
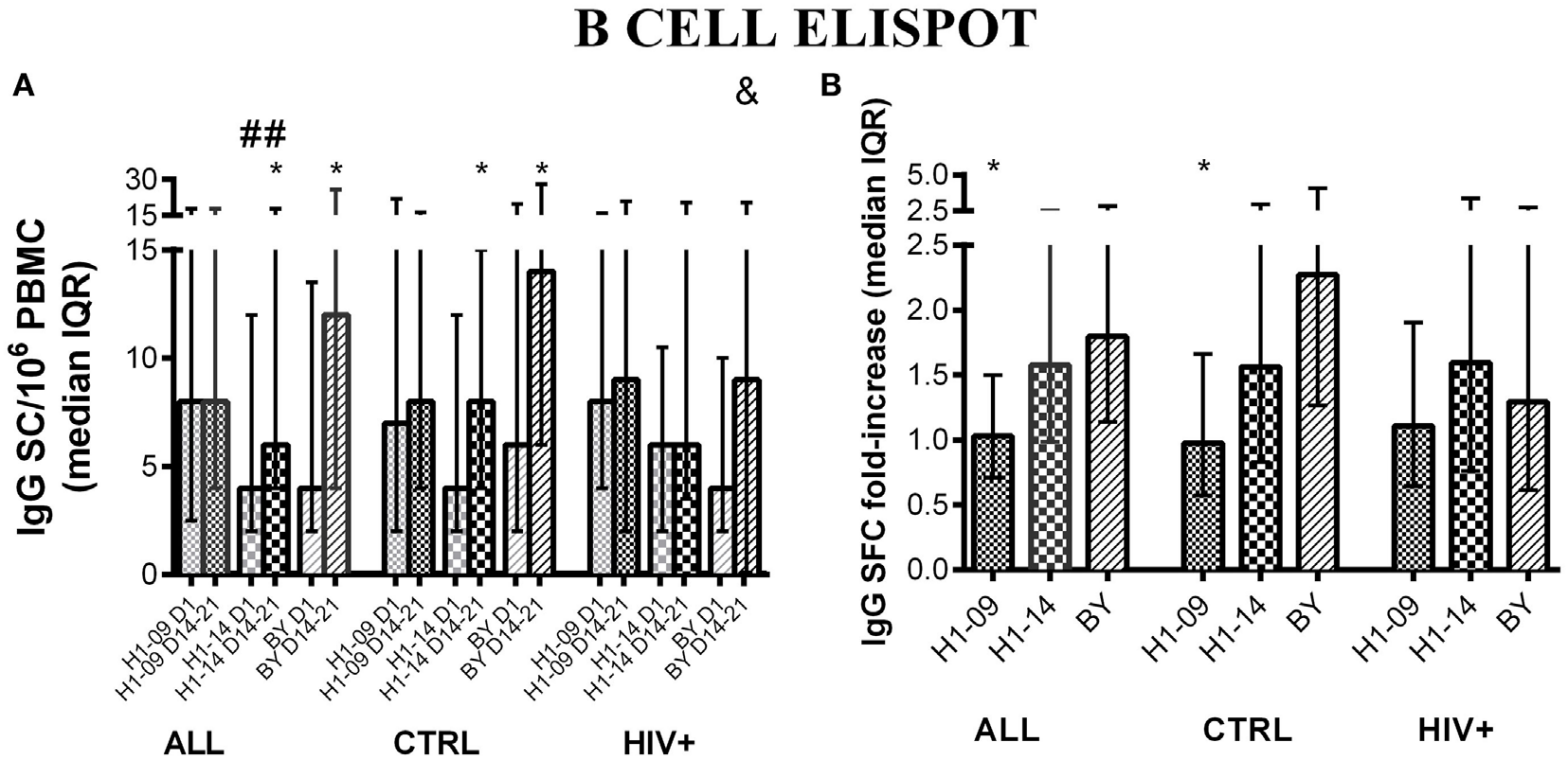
**IgG memory B-cell responses to LAIV4 measured by ELISPOT**. The data were derived from 45 HIV-infected and 55 uninfected individuals. **(A)** Absolute responses in each group of participants, as indicated below the abscissa, at V1 (before vaccination) and V4 (14–21 days after vaccination) and against the viruses indicated in the labels of the abscissa. Asterisks (*) indicate significant differences between pre- and post-vaccination. Hash tags (#) indicate significant differences between H1N1-09 and H1N1-14. And symbols (&) indicate significant differences between HIV-infected and uninfected participants. **(B)** Fold-increases from pre- to post-vaccination. Asterisks (*) indicated significant differences between H1N1-09 and BY.

Before vaccination, IgG B-cell memory responses measured by FluoroSpot were significantly higher for H1N1-09 compared with H1N1-14 across all participants [median (IQR) of 8 (4; 16) vs. 4 (2; 11) IgG SC/10^6^ PBMC; *p* = 0.002; Figure 2A]. Vaccination significantly increased H1N1-14 (*p* = 0.007) but not H1N1-09 IgG memory B cells across all participants, but responses remained higher for H1N1-09 compared with H1N1-14 at visit 4, 14–21 days after vaccination [median (IQR) of 8 (4; 18) vs. 6 (4; 18) IgG SC/10^6^ PBMC; *p* = 0.04; Figure 2A]. IgG B-cell memory to BY significantly increased from pre- to post-vaccination (14–21 days) across all participants [median (IQR) of 4 (2; 14) vs. 12 (4; 26); *p* < 0.0001; Figure 2A]. IgG memory B cell fold-increase from pre- to post-vaccination was significantly higher for BY compared with H1N1-09 (*p* = 0.007; Figure 2B).

HIV status was not associated with any differences in the proportion of IgG memory B cells before or 14–21 days after vaccination for either of the H1N1 strains. At entry, BY-specific IgG SC were similar in HIV-infected and uninfected participants, but 14–21 days after vaccination IgG SC of HIV-infected were lower than those of uninfected vaccinees [median (IQR) of 9 (0; 21) vs. 14 (6; 28) IgG SC/10^6^ PBMC; *p* = 0.04; Figure 2A]. Age did not have a significant effect on IgG memory responses to H1N1 or BY (not depicted).

Most of the participants did not have detectable IgA memory B cells before or after vaccination for any of the influenza strains regardless of age and HIV status. This precluded any meaningful statistical analyses.

### HAI Antibody Responses to H1N1-09, H1N1-14, and BY

**Figure 3:**
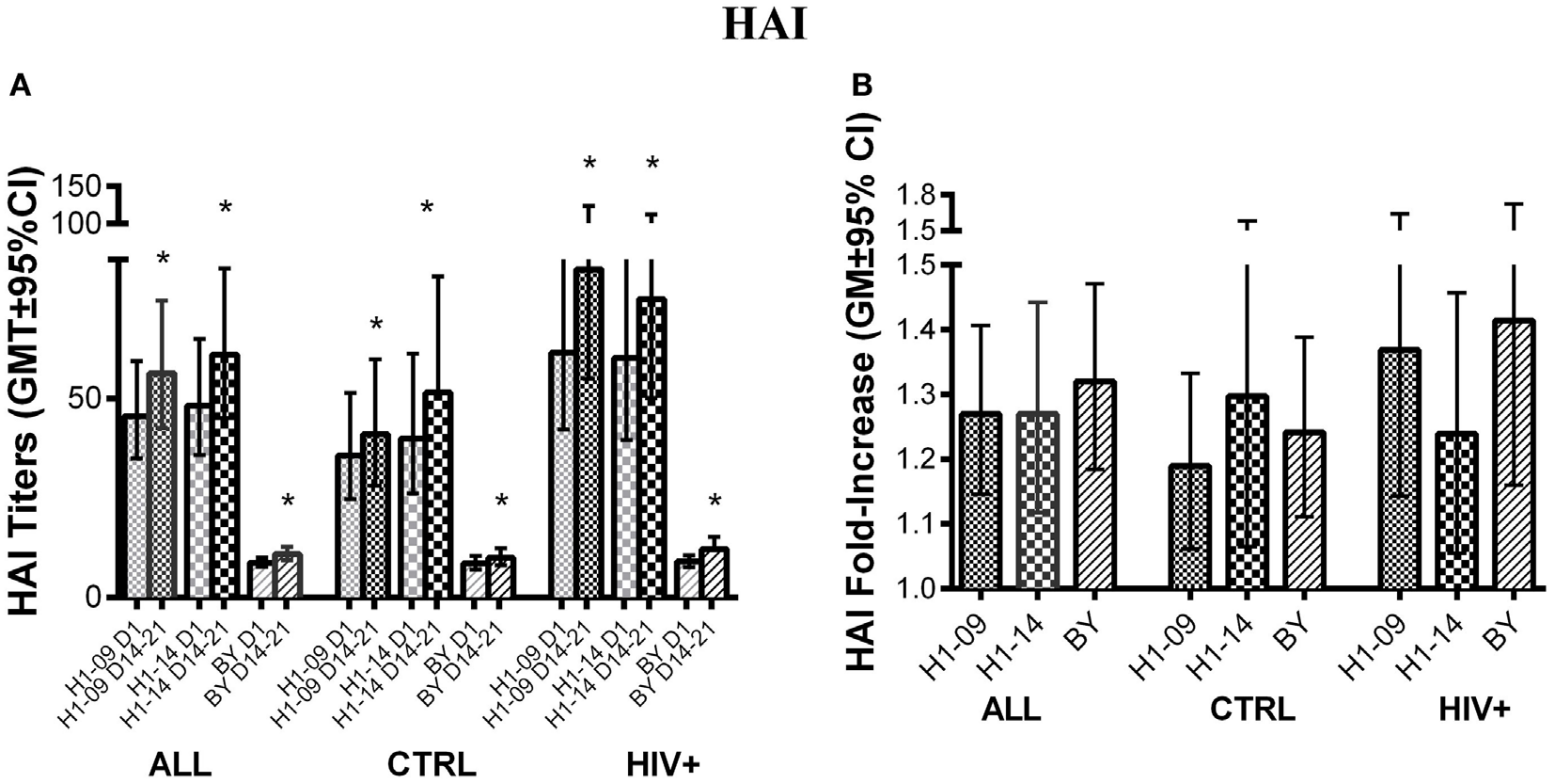
**Antibody responses to LAIV4 measured by HAI**. The data were derived from 45 HIV-infected and 55 uninfected individuals. **(A)** Absolute responses in each group of participants, as indicated below the abscissa, at V1 (before vaccination) and V4 (14–21 days after vaccination) and against the viruses indicated in the labels of the abscissa. Asterisks (*) indicate significant differences between pre- and post-vaccination. **(B)** Fold-increases from pre- to post-vaccination. There were no significant differences across viruses or by HIV status.

Before vaccination, HAI titers did not significantly differ between H1N1-09 and H1N1-14 across all participants [GMT (95% CI) of 45 (35; 59) vs. 48 (36; 65); Figure 3A]. At visit 4 after vaccination (14–21 days), HAI titers similarly increased against both strains of H1N1 (*p* ≤ 0.02; Figures 3A,B). BY-specific HAI titers significantly increased from pre- to 14–21 days post-vaccination [GMT (95% CI) of 8.8 (7.7; 10) to 10.9 (9.4; 12.7), *p* = 0.0002; Figure 3A]. The fold-increases in titers were similar for BY compared with H1N1-09 (Figure 3B).

Hemagglutination inhibition titers pre- and 14–21 days post-vaccination did not differ by HIV status for any of the three influenza strains studied. Age <9 years was associated with lower baseline BY-specific HAI titers and fold-increase (*p* = 0.0003 and 0.04, respectively; not depicted).

### Neutralizing Antibody Responses to H1N1-09 and H1N1-14

**Figure 4:**
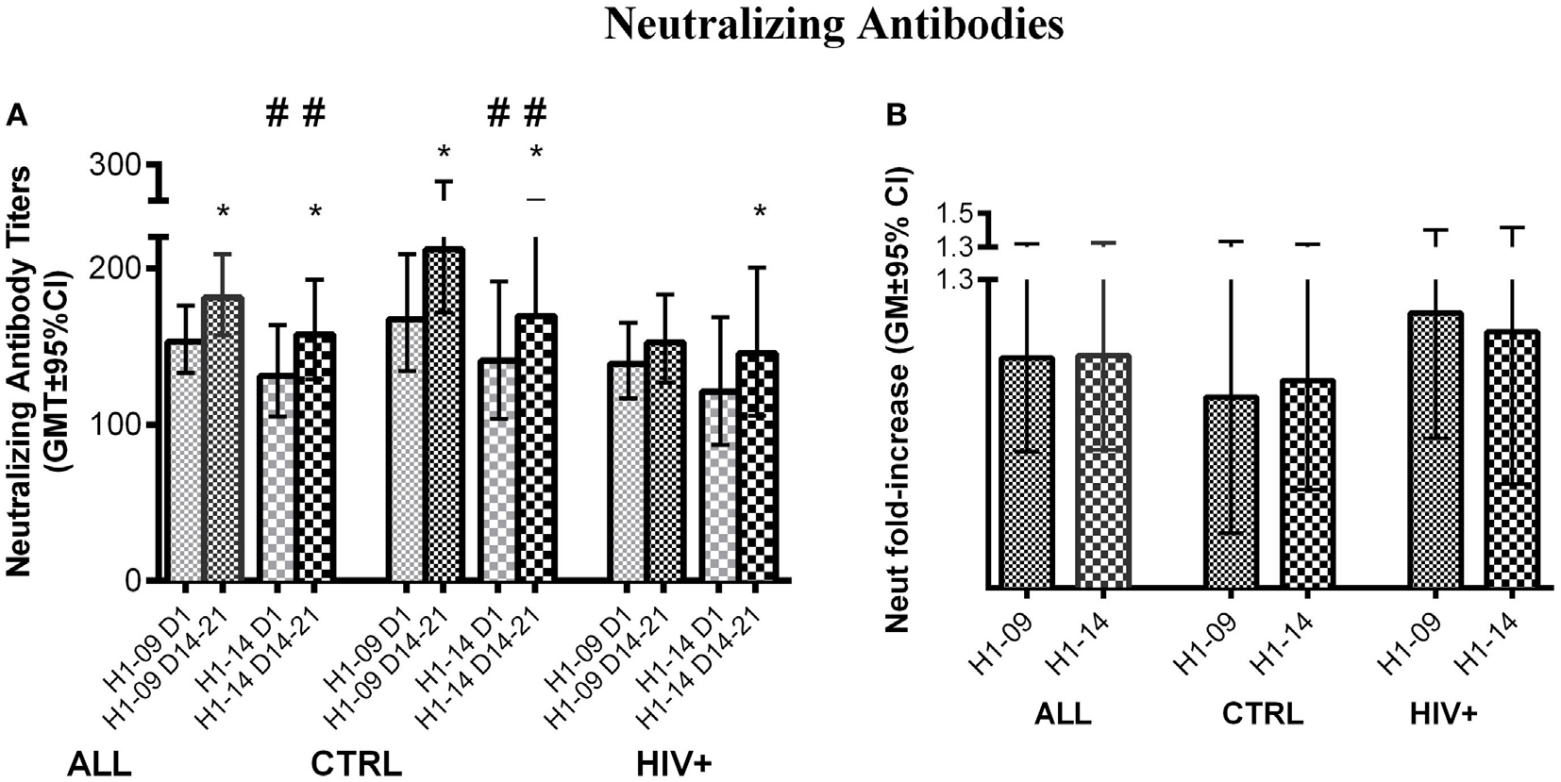
**Neutralizing antibody responses to LAIV4**. The data were derived from 45 HIV-infected and 55 uninfected individuals. **(A)** Absolute responses in each group of participants, as indicated below the abscissa, at V1 (before vaccination) and V4 (14–21 days after vaccination) and against the viruses indicated in the labels of the abscissa. Asterisks (*) indicate significant differences between pre- and post-vaccination. Hash tags (#) indicate significant differences (*p* < 0.05) and strong trends (0.05 ≤ *p* < 0.1) between H1N1-09 and H1N1-14. **(B)** Fold-increases from pre- to post-vaccination.

Before vaccination, neutralizing antibody titers were higher for H1N1-09 than H1N1-14 across all participants [GMT (95% CI) of 153 (133; 176) vs. 131 (105; 164); *p* = 0.03; Figure 4A] and in HIV-uninfected participants [GMT (95% CI) of 168 (134; 209) vs. 121 (87; 169); *p* = 0.04; Figure 4A]. Neutralizing titers against both H1N1 strains significantly increased at visit 4 (14–21 days after vaccination) regardless of HIV status or age (*p* ≤ 0.04; Figure 4B). However, after vaccination, titers against H1N1-09 were marginally higher overall and significantly higher in HIV-uninfected vaccinees (*p* of 0.06 and 0.02, respectively; Figure 4A). Age did not have an effect on neutralizing antibody titers.

### Nasal IgA Antibody Responses to H1N1-09, H1N1-14, and BY

**Figure 5:**
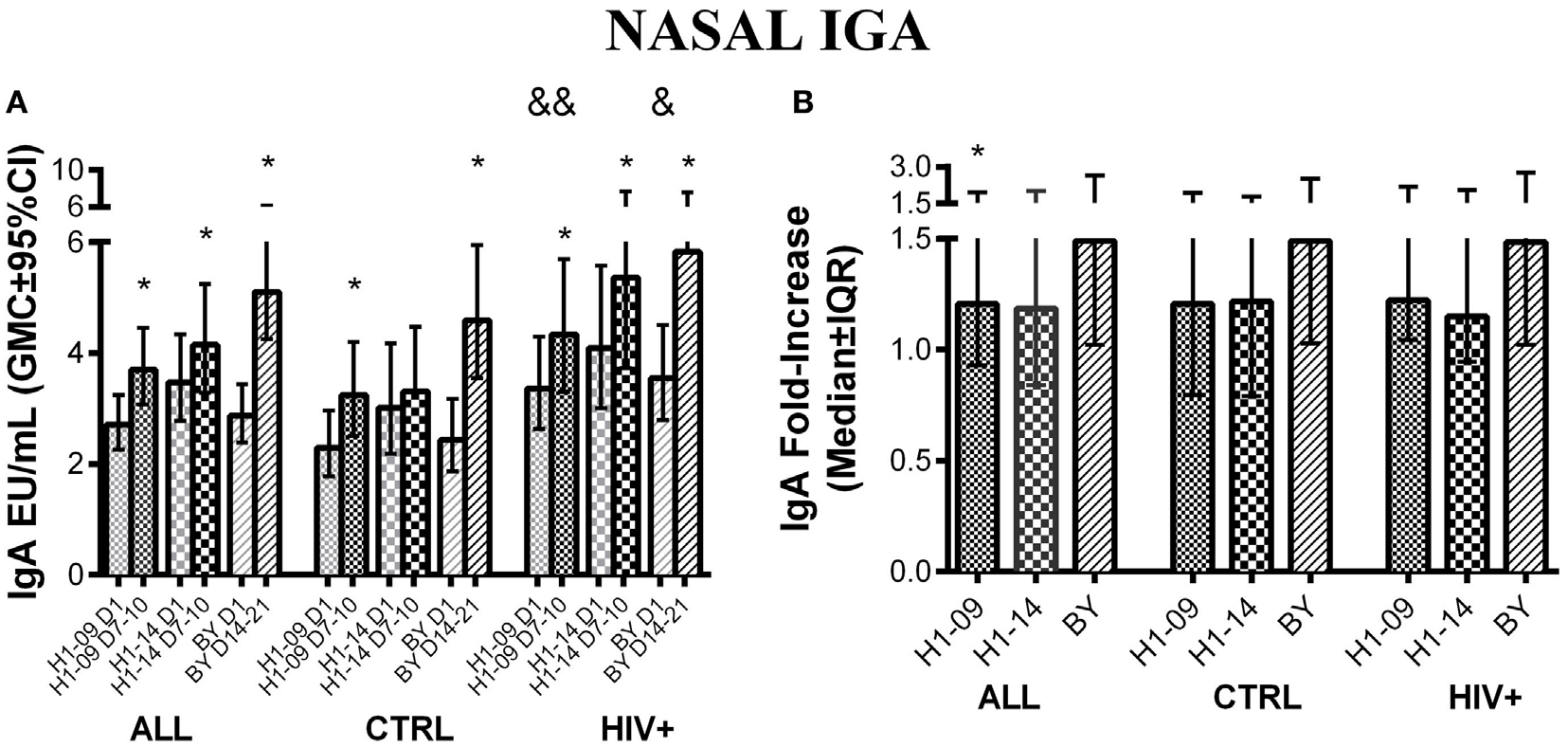
**Nasal IgA antibody responses to LAIV4 measured by ELISA**. The data were derived from 45 HIV-infected and 55 uninfected individuals. **(A)** Absolute responses in each group of participants, as indicated below the abscissa, at V1 (before vaccination) and at the maximum response, which was V3 (7–10 days after vaccination) for H1N1 viruses and V4 (14–21 days after vaccination) for BY. The viruses are indicated in the labels of the abscissa. Asterisks (*) indicate significant differences between pre- and post-vaccination. And symbols (&) indicate significant differences between HIV-infected and uninfected participants. **(B)** Fold-increases from pre- to post-vaccination. Asterisks (*) indicated significant differences between H1N1-09 and BY.

Overall, before vaccination, influenza-specific IgA concentrations in nasal secretions were similar between H1N1-09 and H1N1-14 and significantly increased from pre- to post-vaccination with a peak at visit 3, 7–10 days after vaccination [GMC (95% CI) of 2.3 (2.6; 3.3) to 3.7 (3.1; 4.5), *p* < 0.0001; and 3.5 (2.8; 4.3) vs. 4.2 (3.3; 5.2), *p* = 0.01, respectively; Figure 5A]. BY IgA significantly increased from baseline to post-vaccination reaching its peak at visit 4, 14–21 days after vaccination [GMC (95% CI) of 2.4 (1.9; 3.2) to 4.6 (3.5; 5.9), *p* < 0.0001; Figure 5A]. IgA fold-increases from pre- to post-vaccination were similar for H1N1-09 and H1N1-14, but were significantly higher for BY compared with H1N1-09 across all participants (*p* = 0.005; Figure 5B).

There were no significant differences in nasal IgA concentrations against H1N1 or BY strains by HIV status. Age <9 years was significantly associated with lower fold-increases and post-vaccination peak concentrations to BY (*p* ≤ 0.009; not depicted).

## Discussion

In this first comparison of the immunogenicity of LAIV4 administered in parallel to HIV-infected and uninfected children and youth 2–25 years of age, we showed that HIV-infected vaccinees with average CD4 cell numbers >500 cells/μl and low HIV replication on cART mounted cellular, humoral, and mucosal responses comparable to those of uninfected controls. No differences were observed in the HAI, neutralizing and mucosal IgA or in the T-cell responses to LAIV4. Compared with uninfected controls, HIV-infected participants had lower T-cell immunity to H1N1-09 before vaccination and because the vaccine did not elicit a T-cell response to H1N1-09, the difference persisted after vaccination. By contrast, T-cell immunity to BY, which was also lower at baseline in HIV-infected compared with uninfected participants, significantly increased after vaccination removing any differences. The significance of the low baseline T-cell immunity against the influenza vaccine viruses in HIV-infected individuals compared with uninfected controls needs to be further elucidated. Since 80% of the HIV-infected and 78% of the uninfected participants received IIV in the previous influenza season and there were no differences in baseline HAI titers by HIV status, the difference in T-cell immunity might reflect lower T-cell responses to IIV in HIV-infected compared with uninfected vaccinees and/or accelerated loss of the T-cell immunity in HIV-infected children and youth.

Although most immune responses to LAIV4 had similar magnitude in HIV-infected and uninfected participants, probably as a result of the immune reconstitution or preservation and low viral replication on cART, residual defects persisted in the memory B-cell responses. Only the BY component of LAIV4 generated significant increases in IgG memory B cells. At entry, the IgG memory B-cell immunity to BY was similar in HIV-infected compared with uninfected groups, but after vaccination, HIV-infected participants had lower IgG memory B-cell immunity to the BY. This finding is in accordance with previous reports, indicating that HIV-infected individuals maintain B-cell functional and/or phenotypic defects even after other immune responses improve as a result of cART (28, 29). Memory B-cell responses to influenza were shown to play an important role for heterotypic cross-reactivity (30). Therefore, in years where the vaccine and circulating strains of influenza are well matched, this might not be problematic.

We compared the overall immunogenicity of BY and H1N1 components of LAIV4, because differences in the immunogenicity of the vaccine viruses had the potential to introduce biases in the primary outcome analyses as discussed above. The data showed lower T cell and nasal IgA responses to H1N1 compared with BY. To minimize assay variability, all the viruses and antigens used in the ELISPOT and ELISA contained similar numbers of HAU and all samples of each participant were assayed against all viruses in the same run. In general, low baseline immunologic variables have been associated with higher fold-increases after vaccination (21). This may have contributed to the differences observed in the IgA responses, but not in ELISPOT responses where baseline results were higher for BY compared to H1N1.

Age is an important determinant of responses to influenza vaccines such that it is recommended that children under the age of 9 years to receive 2 doses of influenza vaccines when immunized for the first time. In this study, HIV-infected participants were significantly older than uninfected controls. This difference was at least partially mitigated by requiring all participants to have a history of influenza vaccination in ≥1 year prior to study entry. Nevertheless, we found that age <9 years was associated with decreased nasal IgA and HAI responses to BY. The B Massachusetts 2012 BY was first introduced into the vaccine in 2013/2014, which is likely to have contributed to the lower baseline responses in children <9 years of age. However, our data revealed that children <9 years also had decreased responses to the newly introduced vaccine component in spite of vaccination against influenza in previous years. This novel piece of information has to be further confirmed.

In the 2013–2014 influenza season, H1N1 became again the predominant strain to cause symptomatic disease, hospitalization, and death (31) after having circulated and being included in influenza vaccines since 2009, which suggested that the immune memory raised by H1N1-09 infection or vaccination might not have conferred optimal protection against the circulating H1N1-14. Compared with the H1N1-09 vaccine virus, the H1N1-14 accumulated an equal number of mutations with that observed in H3N2, although in the case of H3N2 the mutations were associated with 2 antigenic drifts (18). Furthermore, a mutation (K166Q) located in a B-cell epitope targeted by human neutralizing antibodies (19) was present in the overwhelming majority of the H1N1 strains circulating in 2013–2014 and also in the clinical H1N1-14 isolate that we used in our assays. In support of the hypothesis that the antigenic differences between H1N1-09 and H1N1-14 were immunologically relevant, our data showed significantly lower immune responses before vaccination against H1N1-14 compared to H1N1-09 mediated by neutralizing antibodies, T cells and IgG memory B cells. While the differences in antibody and memory B-cell responses might be explained by the K166Q mutation in the HA, it is unlikely that this mutation also accounted for the T-cell responses differences. T cell recognize short epitopes, more frequently located in the nucleocapsid proteins than in the HA. Therefore, we would have to assume that H1N1-09 and H1N1-14 differed in other peptides in addition to the HA. It is also notable that the neutralizing antibody titers were affected by the HA mutation, but the HAI titers were not. This suggests a higher specificity of the neutralizing compared with hemagglutination assay. The differences between the H1N1-09 and BY strains persisted after vaccination, either because of the low immunogenicity of LAIV4 against H1N1, as in the case of T- and memory B-cell immunity, or in spite of the responses generated by the vaccine, as in the case of the neutralizing antibodies. Taken together, the data suggest that the antigenic differences between H1N1-09 and H1N1-14 might have further contributed to the low effectiveness of LAIV4 against H1N1 in 2013–2014.

Of note, the HAI titers before or after vaccination did not differ between H1N1-09 and H1N1-14, and the fold-increases also did not differ by vaccine virus, HIV status, or age. In contrast to ELIPOT, neutralization and nasal IgA detected ≥1 differences in the responses to LAIV4 across hosts or viruses. This may be specific to LAIV among all influenza vaccines, because HAI responses generated by LAIV tend to be lower than those generated by subunit vaccines. However, it is important to follow up on this observation in additional studies, because HAI is currently the benchmark for evaluating new influenza vaccines.

Of most important clinical significance, we found that LAIV4 was equally immunogenic in HIV-infected children with high CD4 cell numbers and low viral replication on cART as in uninfected children with respect to T-cell-mediated and mucosal IgA responses. These immune response categories were shown to correlate with the LAIV-conferred protection (8–10). This indicates that LAIV is likely to have similar effectiveness in HIV-infected children with immune preservation or reconstitution on cART and uninfected children. Another clinically important finding was that children <9 years of age, despite past influenza immunization, had decreased responses to a strain of influenza newly introduced into the vaccine.

## Author Contributions

AW and DC designed the study. DC and MN created the study-specific database, enrolled the participants and collected clinical samples and information. MN, DJC, EJ, JP, and DI performed the laboratory assays. AW, CA, and DF performed the data analysis and interpretation of results. AW wrote the manuscript. All authors reviewed, commented, and approved the manuscript.

## Conflict of Interest Statement

The authors do not have a commercial or other association that might pose a conflict of interest.

## Footnotes

^1^ https://www.cdc.gov/flu/professionals/acip/immunogenicity.htm

^2^ http://www.cdc.gov/vaccines/acip/meetings/downloads/min-archive/min-2015-02.pdf

## References

[1] Centers for Disease Control and Prevention (CDC). Estimates of deaths associated with seasonal influenza – United States, 1976-2007. MMWR Morb Mortal Wkly Rep (2010) 59:1057–62. 20798667

[2] Belshe RB Gruber WC Mendelman PM Cho I Reisinger K Block SL Efficacy of vaccination with live attenuated, cold-adapted, trivalent, intranasal influenza virus vaccine against a variant (A/Sydney) not contained in the vaccine. J Pediatr (2000) 136:168–75. 10.1016/S0022-3476(00)70097-7 10657821

[3] Stiver HG Graves P Eickhoff TC Meiklejohn G. Efficacy of “Hong Kong” vaccine in preventing “England” variant influenza A in 1972. N Engl J Med (1973) 289:1267–71. 10.1056/NEJM197312132892402 4127183

[4] Ambrose CS Wu X Caspard H Belshe RB. Efficacy of live attenuated influenza vaccine against influenza illness in children as a function of illness severity. Vaccine (2014) 32:5546–8. 10.1016/j.vaccine.2014.07.097 25131746

[5] Belshe RB Edwards KM Vesikari T Black SV Walker RE Hultquist M Live attenuated versus inactivated influenza vaccine in infants and young children. N Engl J Med (2007) 356:685–96. 10.1056/NEJMoa065368 17301299

[6] Ambrose CS Levin MJ Belshe RB. The relative efficacy of trivalent live attenuated and inactivated influenza vaccines in children and adults. Influenza Other Respir Viruses (2011) 5:67–75. 10.1111/j.1750-2659.2010.00183.x 21306569 PMC3151550

[7] Lanthier PA Huston GE Moquin A Eaton SM Szaba FM Kummer LW Live attenuated influenza vaccine (LAIV) impacts innate and adaptive immune responses. Vaccine (2011) 29:7849–56. 10.1016/j.vaccine.2011.07.093 21816194 PMC3757566

[8] Forrest BD Pride MW Dunning AJ Capeding MR Chotpitayasunondh T Tam JS Correlation of cellular immune responses with protection against culture-confirmed influenza virus in young children. Clin Vaccine Immunol (2008) 15:1042–53. 10.1128/CVI.00397-07 18448618 PMC2446637

[9] Belshe RB Gruber WC Mendelman PM Mehta HB Mahmood K Reisinger K Correlates of immune protection induced by live, attenuated, cold-adapted, trivalent, intranasal influenza virus vaccine. J Infect Dis (2000) 181:1133–7. 10.1086/315323 10720541

[10] Ambrose CS Wu X Jones T Mallory RM. The role of nasal IgA in children vaccinated with live attenuated influenza vaccine. Vaccine (2012) 30:6794–801. 10.1016/j.vaccine.2012.09.018 23000125

[11] King JC Jr Treanor J Fast PE Wolff M Yan L Iacuzio D Comparison of the safety, vaccine virus shedding, and immunogenicity of influenza virus vaccine, trivalent, types A and B, live cold-adapted, administered to human immunodeficiency virus (HIV)-infected and non-HIV-infected adults. J Infect Dis (2000) 181:725–8. 10.1086/315246 10669363

[12] Levin MJ Song LY Fenton T Nachman S Patterson J Walker R Shedding of live vaccine virus, comparative safety, and influenza-specific antibody responses after administration of live attenuated and inactivated trivalent influenza vaccines to HIV-infected children. Vaccine (2008) 26:4210–7. 10.1016/j.vaccine.2008.05.054 18597900 PMC2615200

[13] Pass RF Nachman S Flynn PM Muresan P Fenton T Cunningham CK Immunogenicity of licensed influenza A (H1N1) 2009 monovalent vaccines in HIV-infected children and youth. J Pediatric Infect Dis Soc (2013) 2:352–60. 10.1093/jpids/pit040 24363932 PMC3869470

[14] Flynn PM Nachman S Muresan P Fenton T Spector SA Cunningham CK Safety and immunogenicity of 2009 pandemic H1N1 influenza vaccination in perinatally HIV-1-infected children, adolescents, and young adults. J Infect Dis (2012) 206:421–30. 10.1093/infdis/jis360 22615311 PMC3490699

[15] Madhi SA Dittmer S Kuwanda L Venter M Cassim H Lazarus E Efficacy and immunogenicity of influenza vaccine in HIV-infected children: a randomized, double-blind, placebo controlled trial. AIDS (2013) 27:369–79. 10.1097/QAD.0b013e32835ab5b2 23032417

[16] Abzug MJ Nachman SA Muresan P Handelsman E Watts DH Fenton T Pediatric adolescent, safety and immunogenicity of 2009 pH1N1 vaccination in HIV-infected pregnant women. Clin Infect Dis (2013) 56:1488–97. 10.1093/cid/cit057 23378284 PMC3634309

[17] Faix DJ Hawksworth AW Myers CA Hansen CJ Ortiguerra RG Halpin R Decreased serologic response in vaccinated military recruits during 2011 correspond to genetic drift in concurrent circulating pandemic A/H1N1 viruses. PLoS One (2012) 7:e34581. 10.1371/journal.pone.0034581 22514639 PMC3326053

[18] Klein EY Serohijos AW Choi JM Shakhnovich EI Pekosz A. Influenza A H1N1 pandemic strain evolution – divergence and the potential for antigenic drift variants. PLoS One (2014) 9:e93632. 10.1371/journal.pone.0093632 24699432 PMC3974778

[19] Linderman SL Chambers BS Zost SJ Parkhouse K Li Y Herrmann C Potential antigenic explanation for atypical H1N1 infections among middle-aged adults during the 2013-2014 influenza season. Proc Natl Acad Sci USA (2014) 111:15798–803. 10.1073/pnas.1409171111 25331901 PMC4226110

[20] Yasugi M Kubota-Koketsu R Yamashita A Kawashita N Du A Misaki R Emerging antigenic variants at the antigenic site Sb in pandemic A(H1N1)2009 influenza virus in Japan detected by a human monoclonal antibody. PLoS One (2013) 8:e77892. 10.1371/journal.pone.0077892 24147093 PMC3797713

[21] Curtis D Ning MF Armon C Li S Weinberg A. Safety, immunogenicity and shedding of LAIV4 in HIV-infected and uninfected children. Vaccine (2015) 33:4790–7. 10.1016/j.vaccine.2015.07.082 26241950

[22] Weinberg A Song LY Walker R Allende M Fenton T Patterson-Bartlett J Anti-influenza serum and mucosal antibody responses after administration of live attenuated or inactivated influenza vaccines to HIV-infected children. J Acquir Immune Defic Syndr (2010) 55:189–96. 10.1097/QAI.0b013e3181e46308 20581690 PMC3290334

[23] Rowe T Abernathy RA Hu-Primmer J Thompson WW Lu X Lim W Detection of antibody to avian influenza A (H5N1) virus in human serum by using a combination of serologic assays. J Clin Microbiol (1999) 37:937–43. 10074505 10.1128/jcm.37.4.937-943.1999 PMC88628

[24] Hancock K. In: ID Immunology and Pathogenesis Branch, editor. Influenza Virus Microneutralization Assay. Atlanta, GA: Department of Health and Human Services, Centers for Disease Control and Prevention (2011). p. 1–32.

[25] Weinberg A Song LY Wilkening CL Fenton T Hural J Louzao R Optimization of storage and shipment of cryopreserved peripheral blood mononuclear cells from HIV-infected and uninfected individuals for ELISPOT assays. J Immunol Methods (2010) 363:42–50. 10.1016/j.jim.2010.09.032 20888337 PMC3068047

[26] Weinberg A Song LY Wilkening C Sevin A Blais B Louzao R Optimization and limitations of use of cryopreserved peripheral blood mononuclear cells for functional and phenotypic T-cell characterization. Clin Vaccine Immunol (2009) 16:1176–86. 10.1128/CVI.00342-08 19515870 PMC2725535

[27] Bull M Lee D Stucky J Chiu YL Rubin A Horton H Defining blood processing parameters for optimal detection of cryopreserved antigen-specific responses for HIV vaccine trials. J Immunol Methods (2007) 322:57–69. 10.1016/j.jim.2007.02.003 17382342 PMC1904432

[28] Jacobsen MC Thiebaut R Fisher C Sefe D Clapson M Klein N Pediatric human immunodeficiency virus infection and circulating IgD+ memory B cells. J Infect Dis (2008) 198:481–5. 10.1086/590215 18582200

[29] Levesque MC Moody MA Hwang KK Marshall DJ Whitesides JF Amos JD Polyclonal B cell differentiation and loss of gastrointestinal tract germinal centers in the earliest stages of HIV-1 infection. PLoS Med (2009) 6:e1000107. 10.1371/journal.pmed.1000107 19582166 PMC2702159

[30] Tsang JS Schwartzberg PL Kotliarov Y Biancotto A Xie Z Germain RN Global analyses of human immune variation reveal baseline predictors of postvaccination responses. Cell (2014) 157:499–513. 10.1016/j.cell.2014.03.031 24725414 PMC4139290

[31] Flannery B Thaker SN Clippard J Monto AS Ohmit SE Zimmerman RK Interim estimates of 2013-14 seasonal influenza vaccine effectiveness – United States, February 2014. MMWR Morb Mortal Wkly Rep (2014) 63:137–42. 24553196 PMC4584757

